# The activation of Mucolipin TRP channel 1 (TRPML1) protects motor neurons from L-BMAA neurotoxicity by promoting autophagic clearance

**DOI:** 10.1038/s41598-019-46708-5

**Published:** 2019-07-24

**Authors:** Valentina Tedeschi, Tiziana Petrozziello, Maria José Sisalli, Francesca Boscia, Lorella Maria Teresa Canzoniero, Agnese Secondo

**Affiliations:** 1 Division of Pharmacology, Department of Neuroscience, Reproductive and Odontostomatological Sciences, School of Medicine, “Federico II” University of Naples, Via S.Pansini 5, Napoli, 80131 Italy; 2 Division of Pharmacology, Department of Science and Technology-DST, University of Sannio, via Port’Arsa 11, 82100 Benevento, Italy

**Keywords:** Neuroscience, Molecular neuroscience

## Abstract

Cellular clearance mechanisms including the autophagy-lysosome pathway are impaired in amyotrophic lateral sclerosis (ALS). One of the most important proteins involved in the regulation of autophagy is the lysosomal Ca^2+^ channel Mucolipin TRP channel 1 (TRPML1). Therefore, we investigated the role of TRPML1 in a neuronal model of ALS/Parkinson-dementia complex reproduced by the exposure of motor neurons to the cyanobacterial neurotoxin beta-methylamino-L-alanine (L-BMAA). Under these conditions, L-BMAA induces a dysfunction of the endoplasmic reticulum (ER) leading to ER stress and cell death. Therefore we hypothesized a dysfunctional coupling between lysosomes and ER in L-BMAA-treated motor neurons. Here, we showed that in motor neuronal cells TRPML1 as well as the lysosomal protein LAMP1 co-localized with ER. In addition, TRPML1 co-immunoprecipitated with the ER Ca^2+^ sensor STIM1. Functionally, the TRPML1 agonist ML-SA1 induced lysosomal Ca^2+^ release in a dose-dependent way in motor neuronal cells. The SERCA inhibitor thapsigargin increased the fluorescent signal associated with lysosomal Ca^2+^ efflux in the cells transfected with the genetically encoded Ca^2+^ indicator GCaMP3-ML1, thus suggesting an interplay between the two organelles. Moreover, chronic exposure to L-BMAA reduced TRPML1 protein expression and produced an impairment of both lysosomal and ER Ca^2+^ homeostasis in primary motor neurons. Interestingly, the preincubation of ML-SA1, by an early activation of AMPK and beclin 1, rescued motor neurons from L-BMAA-induced cell death and reduced the expression of the ER stress marker GRP78. Finally, ML-SA1 reduced the accumulation of the autophagy-related proteins p62/SQSTM1 and LC3-II in L-BMAA-treated motor neurons. Collectively, we propose that the pharmacological stimulation of TRPML1 can rescue motor neurons from L-BMAA-induced toxicity by boosting autophagy and reducing ER stress.

## Introduction

Amyotrophic lateral sclerosis (ALS) is a rare neurological disease characterized by neurodegeneration, progressive voluntary muscle wasting and death^1,2^. The genesis can be either familial, accounting for a mere 10% of cases, or sporadic, responsible for the remaining 90% of cases^3^. To date, a large number of genes have been linked to ALS, including Cu/Zn superoxide dismutase (SOD1)^4^, TAR DNA-binding protein (TDP-43)^5^ fused in sarcoma (FUS)^6^, sequestosome 1 (p62/SQSTM1)^7^, optineurin (OPTN)^8^, TANK binding kinase 1 (TBK1)^9^, VAMP-associated protein B (VAPB)^10^, valosin-containing protein (VCP)^11^, ubiquilin 2 (UBQLN2)^12^, alsin (ALS2)^13^, charged multivesicular body protein 2B (CHMP2B)^14^, dynactin (DCTN)^15^, profilin 1 (PFN1)^16^, factor-induced gene 4 (FIG4)^17^, and a hexanucleotide repeat expansion in the gene C9orf72^18^. On the other hand, considerable attention has been recently devoted to the putative role of the cyanobacterial toxin beta-methylamino-L-alanine (L-BMAA) in generating the amyotrophic lateral sclerosis/Parkinson-dementia complex (ALS/PDC), a Guamanian form of the disease^19^. It has been demonstrated that, during the life of ALS/PDC patients, L-BMAA accumulates in their central nervous system (CNS) as free amino acid or integrated in proteins^20^. Furthermore, in either familial or sporadic ALS, protein aggregates have been observed in the brain, suggesting that cellular clearance mechanisms -including the autophagy-lysosome pathway- may be impaired^21^. However, how the dysfunction of the autophagy-lysosome pathway contributes to the pathogenesis of ALS remains unclear. Although defects in autophagy genes may not be considered the direct cause of ALS, most of the ALS-associated genes are involved in the degradative pathways of autophagy^22^. For instance, mutations in p62 account for approximately 1% of ALS cases^23^; they have been reported also in about 2% of FTD (Frontotemporal Dementia) patients and in the most common form of ALS related to C9orf72^24,25^. Therefore, dysfunctional autophagy may exacerbate disease phenotype^26^. This suggests that autophagy and its machinery may be an attractive therapeutic target for the prevention and treatment of ALS-associated neurodegeneration^27–30^. In particular, boosting the efficiency of autophagy could ameliorate the toxic effects of aggregates in ALS^31^. In this respect, the mTORC1 inhibitor rapamycin and the autophagy-inducing agent trehalose are able to improve prognosis in TDP-43 mutant-^29^ and in SOD1 mutant- mice^32^, respectively.

An important ionic regulator of autophagy is the first member of the mammalian mucolipin transient receptor potential (TRP) subfamily named TRPML1 or mucolipin-1, a cation-permeable channel predominantly localized on the membrane of lysosome. It is a non-selective cation channel^33^ mainly involved in Ca^2+^ signaling during lysosomal fusion with other membranes^34^. Interestingly, a deficiency of TRPML1/mucolipin-1 causes mucolipidosis IV, a lysosomal storage disease characterized by neurodegeneration^35–37^. Importantly, TRPML1 activation leads to lysosomal Ca^2+^ release and calcineurin-dependent TFEB nuclear translocation^38^. In addition, it has been recently demonstrated that TRPML1 is also involved in lysosomal biogenesis^39^.

Because of the importance of lysosomal function in ALS, we hypothesized that this channel could be compromised in the disease. We thus investigated whether the pharmacological activation of TRPML1 by its agonist ML-SA1 could exert a neuronal protection by modulating authophagy. Furthermore, since ALS is associated to ER stress^40,41^, the effect of pharmacological stimulation of TRPML1 has been investigated also on the impairment of ER function in primary motor neurons exposed to L-BMAA.

## Results

### TRPML1 co-localizes with the endoplasmic reticulum (ER) in differentiated Mouse Motor Neuron-Like Hybrid Cells (NSC-34)

**Figure 1:**
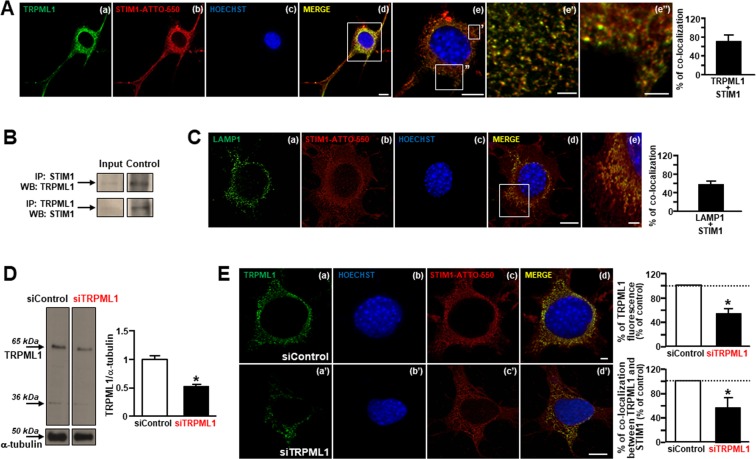
TRPML1 expression and co-localization with the ER Ca^2+^ sensor STIM1 in differentiated NSC-34 cells. (**A**) TRPML1 and STIM1 immunosignals in differentiated NSC-34 cells (a–e). Higher magnifications of the frame “e” illustrate TRPML1/STIM1 staining in cell body (e’–e”). Scale bars: 10 μm (a–d), 5 μm (e), 2 μm (e”) and 1 μm (e’). Bar graph depicts % of TRPML1 and STIM1 co-localization. (**B**) Lysates from differentiated NSC-34 cells subjected to immunoprecipitation (IP) using anti-STIM1 (top row) or anti-TRPML1 (bottom row). (**C**) LAMP1 and STIM1 immunosignals in differentiated NSC-34 cells. Bar graph depicts % of LAMP1 and STIM1 co-localization. Scale bars: 10 μm (a–d), 2 μm (e). (**D**) Representative Western blotting of TRPML1 and α-tubulin expression in NSC-34 cells treated with siControl or siRNA #2 against TRPML1. *p < 0.05 vs siControl. (**E**) TRPML1 and STIM1 immunosignals after transfection with siControl (a–d) or siRNA #2 against TRPML1 (a’–d’). Bar graph on the top depicts the quantification of TRPML1 as fluorescent signal; bar graph on the bottom depicts % of TRPML1 and STIM1 co-localization after siRNAs treatment. Scale bars: 5 μm (a–d), 10 μm (a’–d’). *p < 0.05 vs respective controls.

A tight coupling between ER proteins and lysosomes has been described in many cells^42^. Interestingly, we showed that the main lysosomal Ca^2+^ channel TRPML1 was highly expressed in NSC-34 motor neurons and co-localized with the ER Ca^2+^ sensor STIM1 (Fig. 1A,a–e and Supplementary Fig. S1) as well as with the ER specific probe ER-dsRED (Supplementary Fig. S1). Interestingly, TRPML1 significantly co-immunoprecipitated with STIM1 (Fig. 1B). Furthermore, the high degree of overlap between LAMP1 and STIM1 immunosignals (Fig. 1C,a–e) further confirmed the significant co-localization between lysosomes and ER in NSC-34 motor neuronal cells. Moreover, the channel was efficiently silenced in NSC-34 motor neurons by siRNA strategy (Fig. 1D). Under these conditions, TRPML1 immunosignal was significantly reduced in NSC-34 cells treated with siRNA against the channel (Fig. 1E,a’–d’) compared to the control cells (Fig. 1E,a–d). Furthermore, TRPML1/STIM1 co-localization was significantly reduced in NSC-34 cells treated with siRNA against TRPML1 (Fig. 1E).

**Figure 2:**
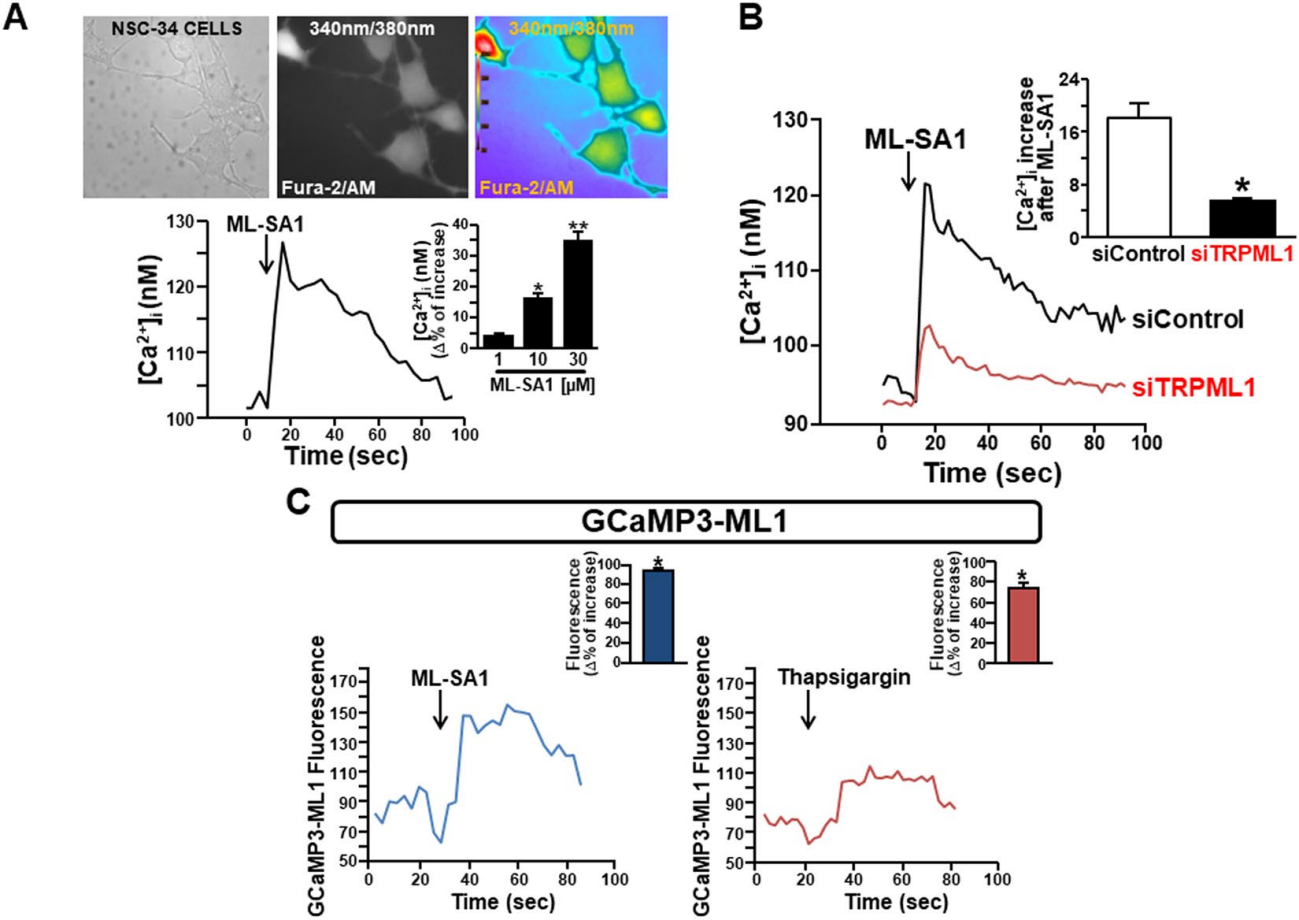
TRPML1 activity in differentiated NSC-34 cells. (**A**) Effect of ML-SA1 (1–30 μM) on [Ca^2+^]_i_ in differentiated NSC-34 motor neurons. Representative trace of the effect of ML-SA1 at 10 μM on [Ca^2+^]_i_ is reported below the images depicting Fura-2-loaded NSC-34 cells as 340 nm/380 nm. Bar graph depicts the dose/effect curve of ML-SA1. Each bar represents the mean ± S.E. (n = 30 cells studied in three different experimental sessions). *p < 0.05 vs control and 1 μM; **p < 0.05 vs 10 μM. (**B**) Superimposed traces representative of the effect of ML-SA1 (10 μM) on [Ca^2+^]_i_ in NSC-34 cells transfected with siControl or siRNA #2 against TRPML1. Inset: bar graph depicts the effect of ML-SA1 expressed as Δ% of [Ca^2+^]_i_ increase. Each bar represents the mean ± S.E. (n = 20 cells studied in three different experimental sessions). *p < 0.05 vs siControl. (**C**) Representative traces of differentiated NSC-34 cells transfected with GCaMP3-ML1 construct and treated with ML-SA1 (10 μM) (left) or thapsigargin (1 μM) (right). Insets: bar graphs depict the quantification of the effect of each drug as % of fluorescence signal increase. Each bar represents drug effects reported as mean ± S.E. (n = 10 cells studied in three different experimental sessions). *p < 0.01 vs respective basal values.

From a functional point of view, a rapid intracellular Ca^2+^ ([Ca^2+^]_i_) increase was detected when NSC-34 motor neurons were exposed to ML-SA1 (1–30 μM) (Fig. 2A), the specific membrane-permeable synthetic agonist of TRPML1 previously characterized by Xu’s group^43^. To further confirm the specificity of the agonist, TRPML1 was silenced in NSC-34 motor neurons. In these cells, ML-SA1 was less effective in determining [Ca^2+^]_i_ increase compared to the control cells (Fig. 2B). To corroborate the specificity of ML-SA1 on TRPML1 activity in motor neurons, lysosome-targeted Ca^2+^ imaging was adopted. ML-SA1 (10 μM) elicited a rapid increase of the fluorescent signal (λ 470 nm) associated with lysosomal Ca^2+^ efflux in NSC-34 cells transfected with GCaMP3-ML1 construct (Fig. 2C, *left*), a single wavelength genetically-encoded Ca^2+^ indicator engineered to the cytoplasmic N-terminus of TRPML1^43^. Interestingly, in a subset of cells, the SERCA blocker thapsigargin (1 μM) produced a large increase of Ca^2+^ signal in NSC-34 transfected with GCaMP3-ML1 construct (Fig. 2C, *right*). These data are in accordance with the hypothesis that ER may serve as primary source of Ca^2+^ for lysosome^44^.

### Lysosomal Ca^2+^ dysregulation is associated with TRPML1 protein downregulation in primary motor neurons exposed to L-BMAA

**Figure 3:**
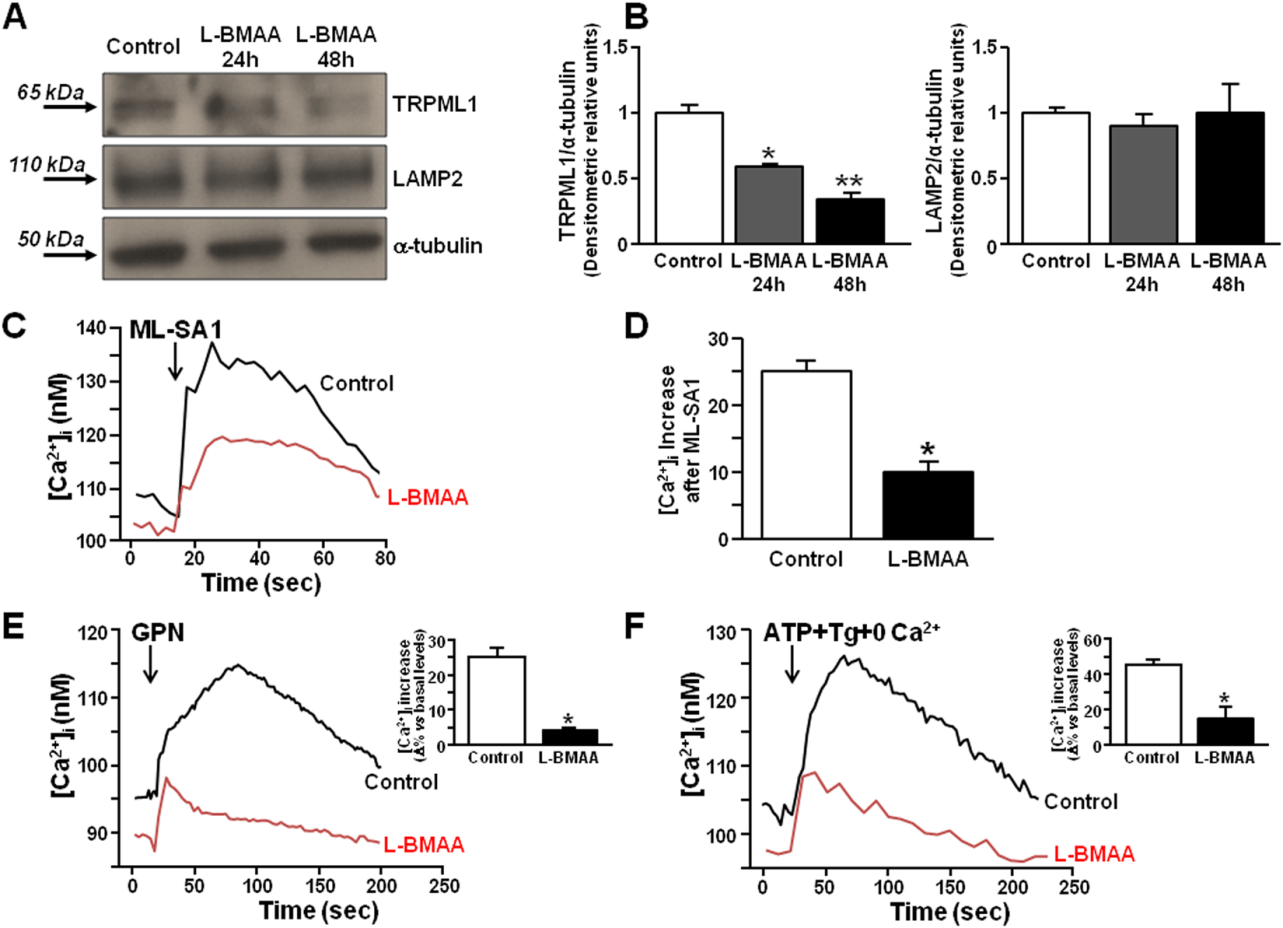
TRPML1 expression and activity in rat primary motor neurons exposed to L-BMAA. (**A**,**B**) Representative Western blotting and quantification of TRPML1 and LAMP2 expression in rat primary motor neurons exposed to L-BMAA (300 μM) for 24 and 48 h. All experiments were repeated at least three times and expressed as mean ± S.E. *p < 0.05 vs control; **p < 0.05 vs 24 h L-BMAA. (**C**,**D**) Representative traces and quantification of the effect of ML-SA1 (10 μM) on [Ca^2+^]_i_ in neurons under control conditions (black trace, n = 20 cells) or after exposure to 48 h L-BMAA (red trace, n = 15 cells). *p < 0.05 vs control. All the experiments were repeated in three different experimental sessions. (**E**,**F**) Representative traces depicting the effect of GPN and ATP + Tg on [Ca^2+^]_i_ in cells treated with the toxin for 48 h. Lysosomal Ca^2+^ content was determined in indirect way by the membrane-permeable dipeptide GPN (300 μM) in a Ca^2+^-containing solution (**E**). ER Ca^2+^ content was determined in indirect way by thapsigargin (Tg, 1 μM) and ATP (100 μM) in 0 Ca^2+^ (**F**). Insets: each bar graph depicts the effect of the treatments in E and F expressed as Δ% of [Ca^2+^]_i_ increase (n = 40 cells in three different experimental sessions). *p < 0.05 vs its respective control. Baseline calcium values in control conditions and in L-BMAA-treated neurons were statistically different (106.9 ± 5 vs 79 ± 6, respectively, *p < 0.001).

Due to the functional coupling between ER and lysosomes under physiological conditions, we have investigated the relationship between these organelles in primary motor neurons chronically exposed to the neurotoxin L-BMAA. To this aim, we studied whether TRPML1-mediated lysosomal Ca^2+^ release could be altered in the proposed neurotoxic model, by detecting the channel protein expression and activity. Figure 3A,B showed that TRPML1 protein expression was progressively downregulated in primary motor neurons exposed to 24 and 48 h of L-BMAA compared to the untreated controls (Fig. 3A,B). On the other hand, the lysosomal marker LAMP2 was not reduced (Fig. 3A,B). Similar results were obtained in differentiated NSC-34 cells (Supplementary Fig. S2).

Then, we investigated whether TRPML1-mediated lysosomal Ca^2+^ release was altered under these conditions. In accordance with the reduced protein expression of TRPML1, ML-SA1 (10 μM) elicited a lower lysosomal Ca^2+^ efflux in primary motor neurons exposed to L-BMAA than in control neurons (Fig. 3C,D). That lysosomal Ca^2+^ content was reduced after L-BMAA exposure was confirmed by the reduction in lysosomal Ca^2+^ discharge elicited by the membrane-permeable dipeptide GPN (300 μM) in motor neurons treated with the toxin (Fig. 3E). Under the same experimental conditions (i.e. 48 h of L-BMAA), a significant ER Ca^2+^ depletion occurred in primary motor neurons, as revealed by the low amount of ER Ca^2+^ released after the addition of ATP + thapsigargin (Fig. 3F). The same results were obtained in differentiated NSC-34 cells (Supplementary Fig. S2).

### TRPML1 dysfunction impairs the autophagic flux in primary motor neurons exposed to L-BMAA

**Figure 4:**
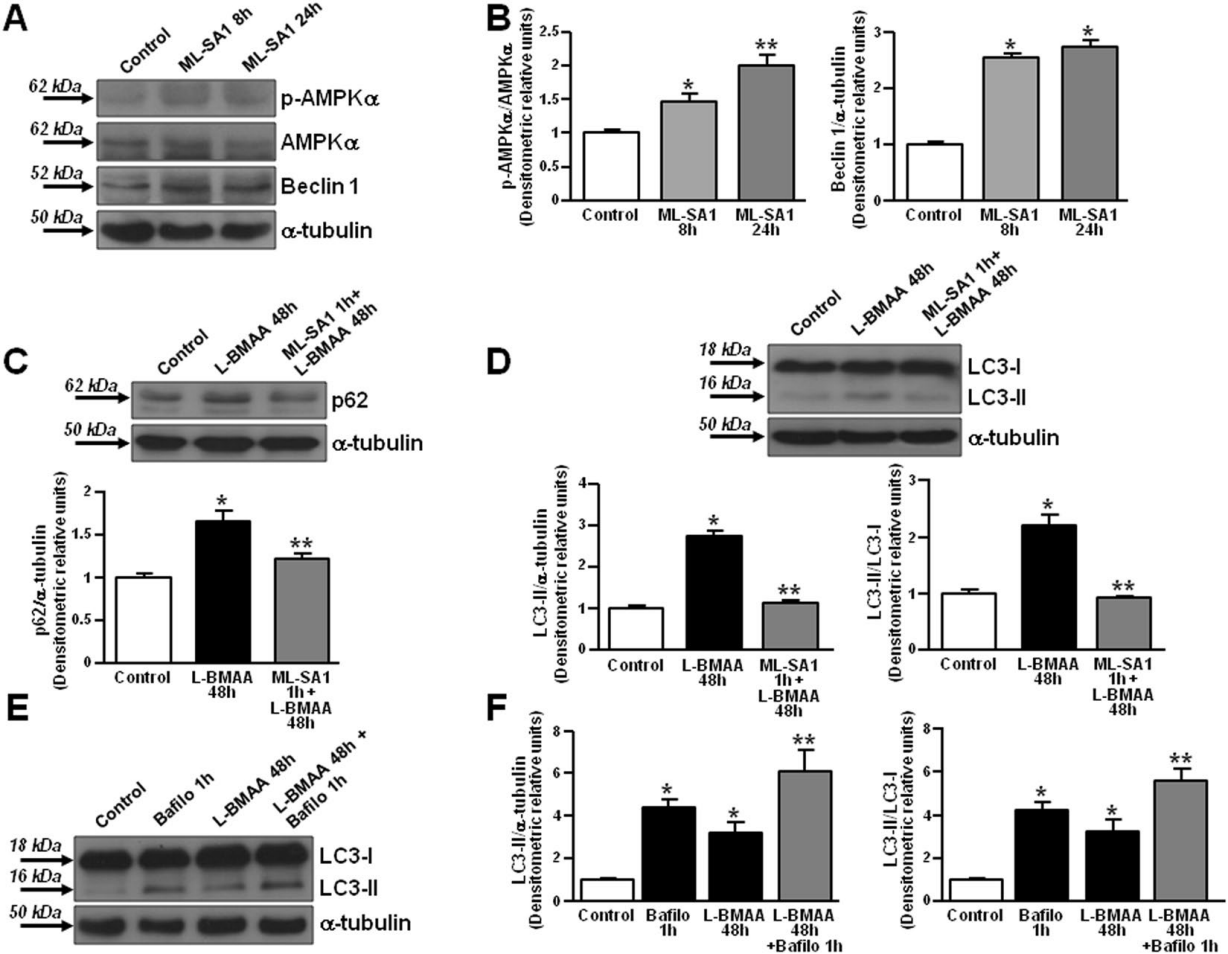
Effect of ML-SA1 on autophagic markers in rat primary motor neurons. (**A**,**B**) Representative Western blotting and quantification of p-AMPKα, AMPKα, and beclin 1 expression in rat primary motor neurons treated for 8 and 24 h with ML-SA1 (10 μM). Each bar represents the mean ± S.E. of data obtained from three different sessions. *p < 0.01 vs control; **p < 0.05 vs control and 8 h ML-SA1. (**C**) Representative Western blotting and quantification of p62 expression in rat primary motor neurons exposed to L-BMAA (300 μM/48 h) in the absence or presence of ML-SA1 (10 μM). Each bar represents the mean ± S.E. of data obtained from three different sessions. *p < 0.01 vs control; **p < 0.05 vs L-BMAA. (**D**) Representative Western blotting and quantification of LC3-I and LC3-II expression in rat primary motor neurons exposed to L-BMAA (300 μM/48 h) in the absence or presence of ML-SA1 (10 μM). Each bar represents the mean ± S.E. of data obtained from three different sessions. *p < 0.01 vs control; **p < 0.05 vs L-BMAA. For (**C**,**D**), ML-SA1 was preincubated for 1 h before L-BMAA exposure. (**E**,**F**) Representative Western blotting and quantification of LC3-I and LC3-II expression in motor neurons exposed to L-BMAA (300 μM/48 h) in the absence or presence of bafilomycin (Bafilo 100 nM/1 h).This latter drug was added 1 h before the end of the experiment. Each bar represents the mean ± S.E. of data obtained from three different sessions. *p < 0.01 vs control; **p < 0.05 vs L-BMAA alone.

Macroautophagy is defective in TRPML1-deficient neurons^45^ whereas TRPML1 overexpression results in a significant increase of the autophagic flux^46^. In order to verify the role of TRPML1 in the modulation of autophagy in primary motor neurons, we tested the effect of ML-SA1 alone on some autophagy initiators. The addition of ML-SA1 progressively induced the phosphorylation of AMP-activated protein kinase (p-AMPK), that is thought to activate autophagy through the inactivation of mTOR complex-1, and increased the expression of beclin 1 (Fig. 4A,B), that is involved in the formation of the autophagosomes. Interestingly, under these conditions, ML-SA1 did not affect fluorescence labeling of axonal neurofilament H detected by NF200 in rat primary motor neurons (in arbitrary units: 20.156 ± 5.824 in control conditions vs 19.193 ± 1.546 in ML-SA1-treated neurons) (Supplementary Fig. S3). On the other hand, to test the hypothesis that TRPML1 reduction could worsen while channel activation could ameliorate autophagy defects in ALS, lysosomal degradation pathway was investigated in this neurotoxic model. L-BMAA (300 μM/48 h) induced a significant increase in the expression of both p62/SQSTM1 (Fig. 4C) and LC3-II (Fig. 4D) that were significantly reduced in the presence of ML-SA1 (Fig. 4C,D). Since bafilomycin further increased LC3-II expression in L-BMAA-treated motor neurons, the exposure to the toxin impaired the autophagic flux in primary motor neurons (Fig. 4E,F).

### TRPML1 activation rescues motor neurons from L-BMAA-induced cell death

**Figure 5:**
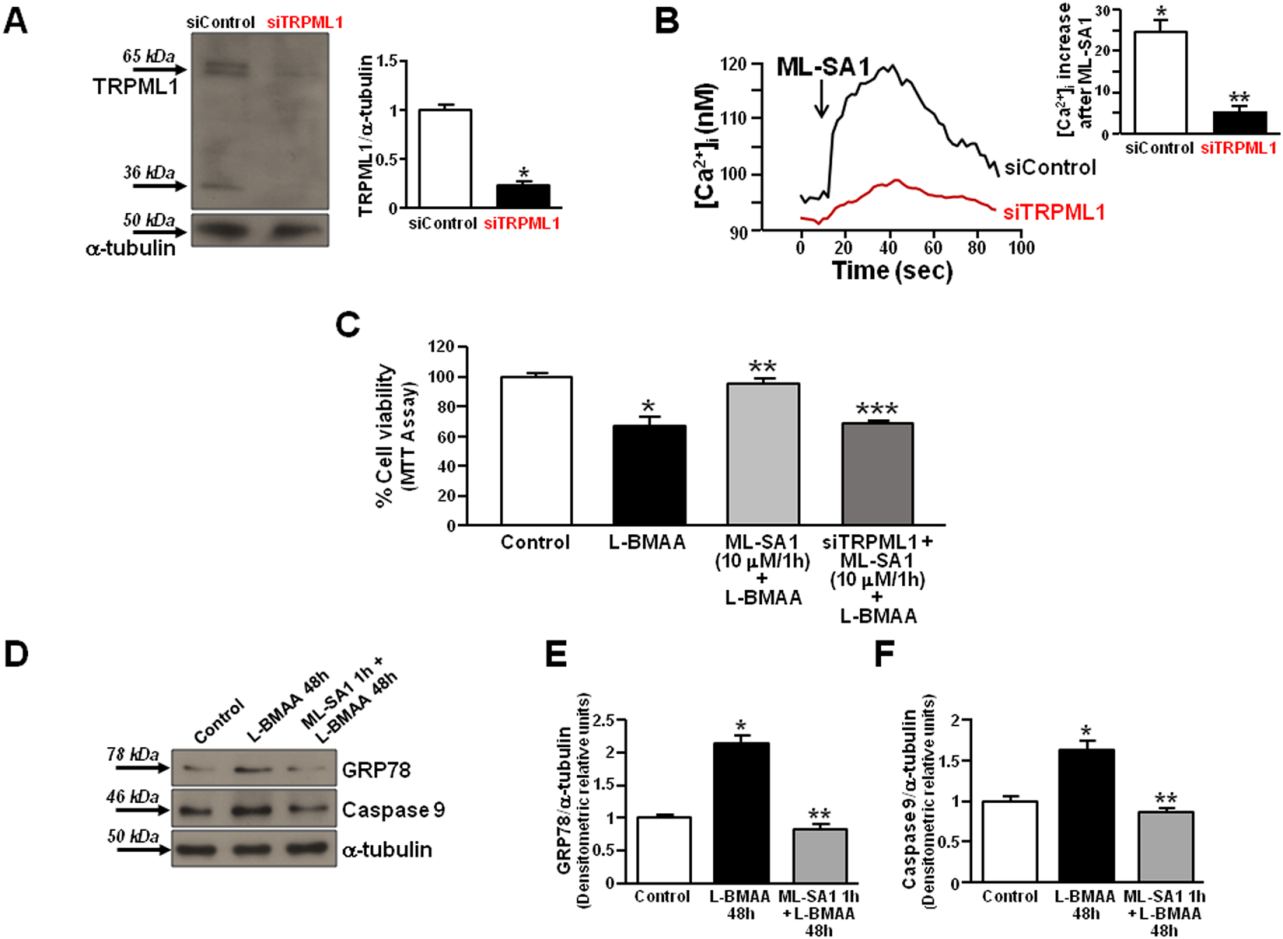
Effect of ML-SA1 on cell death and ER stress in rat primary motor neurons exposed to L-BMAA. (**A**) Representative Western blotting of TRPML1 and α-tubulin expression in rat primary motor neurons treated with siControl or siRNA #2 against TRPML1. *p < 0.05 vs siControl. (**B**) Superimposed traces representative of the effect of ML-SA1 (10 μM) on [Ca^2+^]_i_ in rat primary motor neurons transfected with siControl or siRNA #2 against TRPML1. Inset: bar graph depicts the effect of ML-SA1 expressed as Δ% of [Ca^2+^]_i_ increase. Each bar represents the mean ± S.E. (n = 20 cells studied in three different experimental sessions). *p < 0.05 vs basal values **p < 0.05 vs siControl. (**C**) Bar graph depicting the effect of ML-SA1 on cell viability rate of rat primary motor neurons exposed to L-BMAA (300 μM/48 h) in the presence or absence of siRNA against TRPML1. Data are expressed as mean ± S.E. of three different experimental sessions. *p < 0.05 vs control; **p < 0.05 vs L-BMAA alone; ***p < 0.05 vs ML-SA1 + L-BMAA. (**D**–**F**) Representative Western blotting and quantification of GRP78 (**E**) and caspase 9 (**F**) expression in rat primary motor neurons exposed to L-BMAA (300 μM/48 h) in the absence or presence of ML-SA1 (10 μM). Each bar represents the mean ± S.E. of data obtained from three different sessions. *p < 0.01 vs control; **p < 0.05 vs L-BMAA. For all, ML-SA1 was preincubated for 1 h before L-BMAA exposure.

To study the effect of ML-SA1 on L-BMAA-induced cell death, we tested the effect of this toxin in the absence of TRPML1 channel. TRPML1 protein expression was reduced by 70 ± 1.5% (*p < 0.05 vs control + siControl) in primary motor neurons treated with specific siRNA against the channel (Fig. 5A). Functionally, ML-SA1 (10 μM) determined a significant Ca^2+^ release in primary motor neurons that was significantly reduced in the absence of TRPML1 (Fig. 5B).

Interestingly, the preincubation for 1 h with ML-SA1 (10 μM) reduced L-BMAA-induced cell death in primary motor neurons (Fig. 5C) as well as in differentiated NSC-34 motor neurons, in which the neuroprotective effect occurred also for longer exposures (Supplementary Fig. S2). Importantly, the ML-SA1-induced neuroprotection was counteracted by TRPML1 silencing in primary motor neurons (Fig. 5C). Furthermore, ML-SA1 pretreatment reduced GRP78 and caspase 9 upregulation induced by L-BMAA in primary motor neurons (Fig. 5D–F).

As a consequence of cell suffering avoidance, the preincubation with ML-SA1 prevented TRPML1 protein downregulation induced by chronic exposure to L-BMAA (Supplementary Fig. S3).

## Discussion

**Figure 6:**
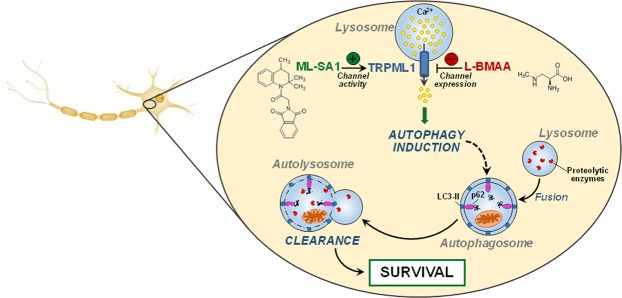
Effect of ML-SA1 on the autophagic flux in L-BMAA-treated motor neurons. Schematic representation of the prosurvival pathway elicited by ML-SA1 involving TRPML1 activation, lysosomal Ca^2+^ release and autophagic flux induction in a neurotoxic model of ALS/PDC.

In recent years, the idea of lysosomes as mere degradative organelles has dramatically changed and they are now recognized as critical regulators of cellular homeostasis and survival. In the present study, we have characterized the involvement of one of the main lysosomal channels, TRPML1, in the pathogenesis of L-BMAA-induced ALS/PDC. We found that TRPML1 Ca^2+^ channel is an important player in the protective response of lysosomes against L-BMAA in primary motor neurons chronically exposed to the neurotoxin. In fact, we provided evidence that TRPML1 was progressively downregulated in L-BMAA-treated neurons while its early stimulation, by the synthetic compound ML-SA1, efficiently rescued motor neurons from L-BMAA toxicity by counteracting ER stress and autophagy impairment (Fig. 6). Furthermore, under these conditions, TRPML1 protein expression was partially restored as a consequence of ML-SA1-induced neuroprotection.

That ML-SA1 stimulated specifically TRPML1 channel was demonstrated by the absence of the effect on Ca^2+^ release in TRPML1-silenced motor neurons. Furthermore, ML-SA1 failed to rescue motor neurons exposed to L-BMAA when TRPML1 was absent. Mechanicistically, lysosomal Ca^2+^ efflux through TRPML1 seems to play a prominent role in the protective action of ML-SA1. In fact, lysosomal Ca^2+^ efflux progressively reduced together with TRPML1 protein expression in motor neurons exposed to the toxin, thus causing lysosomal Ca^2+^ dysfunction and cell death. Furthermore, we showed a concomitant dysfunction in ER Ca^2+^ homeostasis in neurons exposed to L-BMAA, most likely depending on the disruption of the lysosomal and ER functional interplay. Indeed, the present study suggests that ER store acts as a major source of lysosomal Ca^2+^ in motor neurons and that dysfunctional Ca^2+^ homeostasis in one of these organelles has a dramatic repercussion on the other store. This is in accordance with the evidence demonstrating the strategic role of ER in promoting lysosomal Ca^2+^ refilling in other cellular systems^44^. Accordingly, we showed that the specific and irreversible SERCA inhibitor thapsigargin, by depleting ER Ca^2+^ store, influences lysosomal Ca^2+^ content measured as Ca^2+^ release through TRPML1 by the Ca^2+^ indicator GCaMP3-ML1.

Furthermore, in the present study, ML-SA1 prevented the upregulation of the ER stress marker GRP78 that significantly increases after L-BMAA-induced ER store dysfunction^41^ and in sporadic ALS characterized by misfolding of wild-type SOD1^40^. This suggests that TRPML1 stimulation may protect motor neurons from ER stress in different forms of ALS. It could be taken into consideration that, in general, an abundance of ER Ca^2+^ is vital for nascent protein folding and cell survival^47^. Loss of ER luminal Ca^2+^ is frequently accompanied by ER stress and by the activation of an apoptotic pathway. Therefore, ER Ca^2+^ refilling is essential for cell survival under stress conditions^48^. Accordingly, it has been suggested that store-operated calcium entry upon ER calcium depletion is aberrant in SOD1^G93A^ animals^49^. However, our data showed that, in consideration of the close proximity between the two organelles, TRPML1-dependent lysosomal Ca^2+^ release may also contribute to ER Ca^2+^ filling state and to ER stress prevention by a continuous refilling of Ca^2+^ ions. Therefore, we hypothesize that ER and lysosomes co-localization, together with their functional interdependency, can be crucial in the reduction of the neurotoxic effect of L-BMAA.

The toxicity caused by protein misfolding or aggregation, or proteotoxicity, is a common feature of ALS^50^. Ubiquitin-positive inclusions linked to SOD1, TDP-43, FUS, and C9orf72 have been reported in familial ALS^51^. However, abnormal accumulation of the wild-type form of some proteins, such as TDP-43 and SOD1, has also been observed in sporadic ALS^5,40,52^. This is indicative of defects in cellular cleansing mechanisms occurring in the neurodegenerative disease. Given the important role of autophagy in cleansing the cell of defective organelles and misfolded proteins, our data suggest that the activation of TRPML1 could prevent the engulfment in autophagy provoked by chronic exposure to L-BMAA. In fact, TRPML1 stimulation reduced the accumulation of p62/SQSTM1 and LC3-II in motor neurons exposed to the toxin. In this respect, p62 is known to be involved not only in selective autophagy but also in degradation through the ubiquitin-proteasome system^53^. Interestingly, overexpression of p62/SQSTM1 shortens lifespan in a double transgenic ALS mouse model by compromising the protein degradation pathway^54^. On the other hand, Curcio-Morelli and co-authors showed that mouse TRPML1-deficient neurons show a 2-fold increase in p62/SQSTM1 levels, defect in LC3-II clearance, macroautophagy impairment and increase in polyubiquitin levels^45^. This may suggest that TRPML1 is specifically involved in the impairment of the cleansing machinery in ALS.

Recent studies suggest that the aggregation of ALS-related genes can affect early steps in the autophagic process thus producing a hyperactive induction of autophagy suggestive of AMPK activation and mTOR repression in motor neurons of SOD1^G85R^ mice^55^. This determines autophagy engulfment. Consistently, upregulation of beclin 1^56^ has been found in spinal cord and brainstem fractions of SOD1^G93A^ animals in which p62 and LC3-II levels were elevated^57^. The engulfment of autophagy was confirmed by the present study in motor neurons exposed to L-BMAA, since the neurotoxin increased the level of the autophagy markers p62 and LC3-II. However, we showed that TRPML1 stimulation by ML-SA1 prevented the elevation of these markers, thus rescuing the intact autophagic flux.

The therapeutic utility of pharmacological upregulation of autophagy in some neurodegenerative diseases^58^ and in ALS models^59^ is supported by a wealth of data. Nonetheless, enhanced autophagy seems to further exacerbate axonal degeneration and disease phenotype^50,60^. This is indicative of an urgent need for other strategies aimed at finely regulating autophagy to promote neuronal protection and survival. To date, it has been reported that autophagy disturbances vary depending on the phases of disease. In fact, at the early stage of disease, autophagy fulfills a neuroprotective role in SOD^G93A^ mice^61^. The present study suggests that, at least *in vitro*, the initial triggering of lysosomal Ca^2+^ release by TRPML1 stimulation could promote a sort of autophagy reprogramming leading to a long-lasting effect. Altogether, this study shows that the specific membrane-permeable synthetic agonist of TRPML1, ML-SA1, rescued motor neurons from death induced by chronic exposure to L-BMAA. Furthermore, ML-SA1 prevented the elevation of the ER stress marker GRP78 and of the autophagy-related proteins p62/SQSTM1 and LC3-II. Collectively, we propose that an early pharmacological stimulation of lysosomal Ca^2+^ channel TRPML1 can efficiently rescue motor neurons from L-BMAA toxicity through the restoration of the autophagic flux.

## Materials and Methods

### Reagents

Rabbit polyclonal antibodies against TRPML1 (#ACC-081), STIM1-ATTO-550 (#ACC-063-AO) and STIM1 (#ACC-063) were purchased from Alomone Labs (Jerusalem, Israel); mouse monoclonal antibody against α-tubulin (#T5168), rabbit polyclonal anti-neurofilament 200 (NF200) (#N4142), and rabbit polyclonal antibody against LAMP2 (#L0668) were from Sigma-Aldrich (Milan, Italy); rabbit polyclonal antibody against LAMP1 (#AB2971) was from Merck Millipore (Darmstadt, Germany); rabbit polyclonal anti-p-AMPKα (#2531), anti-AMPKα (#2532), and anti-GRP78 (#3183) antibodies were from Cell Signaling Technology, Inc. (Danvers, MA, USA); rabbit polyclonal antibodies against beclin 1 (#NB500-249) and p62 (#NBP1-48320) were from Novus Biologicals (Littleton, CO, USA); rabbit polyclonal antibodies against caspase 9 (#GTX132331) and LC3B (#GTX127375) were from GeneTex Inc. (Irvine, CA, USA). ECL reagents and nitrocellulose membranes were from GE Healthcare (Milan, Italy). siRNAs against TRPML1 and siRNA-control were purchased from Qiagen (Milan, Italy).

2-(2-Oxo-2-(2,2,4-trimethyl-3,4-dihydroquinolin-1(2H)-yl)ethyl)isoindoline-1,3-dione (ML-SA1) was from Merck Millipore (Darmstadt, Germany), while Gly-Phe β-naphthylamide (GPN) was from Santa Cruz Biotechnology, Inc. (Dallas, TX, USA). Retinoic acid, cytosine β-D-arabinofuranoside hydrochloride (Ara-C), L-BMAA, thapsigargin, and bafilomycin A1 were from Sigma-Aldrich (Milan, Italy).

1-[2-(5-Carboxyoxazol-2-yl)-6-aminobenzofuran-5-oxy]-2-(21-amino-51-methylphenoxy)-ethane-N,N,N1,N1-tetraacetic acid penta-acetoxymethyl ester (Fura-2/AM) was from Molecular Probes (Life Technologies, Milan, Italy). DsRed2-ER-5 (ERdsRED) was a kind gift from Dr. Michael Davidson (Addgene plasmid # 55836).

### Use of experimental animals

All the experiments were performed in accordance with relevant guidelines and regulations and with the procedures described in experimental protocols approved by the Ethical Committee of “Federico II” University of Naples, Italy, and by Italian Ministry of Health (D.Lgs. March 4^th^ 2014 from Italian Ministry of Health and DIR 2010/63 from UE).

### Cell cultures

#### Clonal cells

Mouse Motor Neuron-Like Hybrid Cells (NSC-34) were grown in high-glucose Dulbecco’s Modified Eagles Medium (DMEM) supplemented with 10% fetal bovine serum (FBS), 2 mM L-glutamine (Gibco, Thermo Fisher Scientific, Waltham, MA, USA), 100 IU/ml penicillin and 100 μg/ml streptomycin (Sigma-Aldrich, Milan, Italy). Cells were cultured at 37 °C in a humidified atmosphere with 5% CO_2_. Differentiation of NSC-34 cells was promoted by exposing cells to retinoic acid at 10 μM for 48 h, a condition triggering a typical neuronal phenotype^62^.

#### Primary cultures of rat motor neurons

Rat primary motor neurons were isolated from the spinal cord of 12-14-day old Wistar embryos and cultured as previously described^41,63^. To prevent non-neuronal cell growth, cytosine β-D-arabinofuranoside hydrochloride (Ara-C; 10 μM) was added to the cell culture medium at 4 and 8 DIV. These primary cultures were used for experiments at 10-12 DIV.

### L-BMAA and ML-SA1 administration

Motor neurons were exposed to 300 μM L-BMAA for 24 and 48 h. ML-SA1 (10 μM) was added to the cell culture medium 1 h before L-BMAA treatment. The chronic exposure to this non-proteic amino acid has been associated to ALS/PDC, a Guamanian form of ALS affecting Chamorro people^19^.

### Immunocytochemistry and confocal microscopy

Twenty-four hours after transfection with ERdsRED and siTRPML1, NSC-34 cells were washed twice in cold 0.01 M PBS (pH 7.4) and fixed at room temperature in 4% paraformaldehyde for 20 min. Following three washes in PBS, cells were blocked in PBS containing 3% BSA for 30 min and then incubated overnight at 4 °C with anti-TRPML1 antibody (1:1000). In another set of experiments, NSC-34 cells were incubated with anti-TRPML1 (1:1000) or anti-LAMP1 (1:500) antibody and STIM1-ATTO-550 (1:200) (overnight at 4 °C). Then, cells were washed with PBS and incubated with anti-rabbit Cy2-conjugated antibody (1:200, Jackson Immuno Research Laboratories, Inc. PA, USA) for 1 h at room temperature under dark conditions. Cells were finally incubated for 5 min with Hoechst. Controls of the method in the double immunofluorescence experiments included the replacement of primary antibodies against TRPML1 or LAMP1 with normal serum. In addition, the anti-rabbit Cy2-conjugated antibody was highly preadsorbed to the IgGs of numerous species. For immunocytochemical analysis of the image shown in Supplementary Fig. S3, rat primary motor neurons were incubated overnight at 4 °C with anti-neurofilament 200 (NF200) antibody (1:1000). Finally, cover glasses were mounted with a SlowFade^TM^ Antifade Kit (Molecular Probes, Life Technologies, Milan, Italy) and acquired by a 63X oil immersion objective using a Zeiss inverted 700 confocal microscope^64^. Co-localization between TRPML1 or LAMP1 and STIM1-ATTO-550 was analyzed by using the ‘co-localization highlighter’ plug-in for ImageJ Software (NIH, Bethesda, MA, USA). Before the analysis of co-localization, threshold settings for each image were determined, and quantification was accomplished by counting the number of TRPML1 or LAMP1 and STIM1 co-localized points per microscope field. Results were expressed as a percentage of co-localization^64^. Fluorescence intensity of TRPML1 was quantified as pixel intensity value with the NIH image software. Digital images were acquired with 63X objective and the same laser power settings were applied to all the photographs for each experimental set^65^.

### [Ca^2+^]_i_ measurements and GCaMP3-ML1 Ca^2+^ imaging in single living cells

[Ca^2+^]_i_ was detected by single-cell Fura-2/AM computer assisted video-imaging, as previously reported^66^. Results are presented as cytosolic Ca^2+^ concentration calculated by the equation of Grynkiewicz *et al*.^67,68^.

To monitor lysosomal Ca^2+^ efflux through TRPML1, motor neurons were transfected with GCaMP3-ML1 construct in OptiMem medium by HiPerFect Transfection Reagent (Qiagen, Milan, Italy). After 24 h, GCaMP3-ML1 fluorescence intensity was monitored at 470 nm.

GCaMP3-ML1 construct was a generous gift from Dr. Haoxing Xu (Department of Molecular, Cellular, and Developmental Biology, University of Michigan, 3089 Natural Science Building (Kraus), 830 North University, Ann Arbor, MI 48109, USA)^43^.

### Small interfering RNAs

In primary motor neurons TRPML1 knocking-down was achieved by three different FlexiTube small interfering RNAs (siRNAs) against the channel (Qiagen, Milan, Italy). Neurons were transfected in OptiMem medium by HiPerFect Transfection Reagent with the non-targeting control and the following siRNAs (all at 10 nM):

(#1) Rn_LOC288371_4 (sense strand: 5′-GCAGAACGAGUUUGUUGUATT-3′; antisense strand: 5′-UACAACAAACUCGUUCUGCAG-3′),

(#2) Rn_LOC288371_3 (sense strand: 5′-UGAUCACAUUUGACAAUAATT-3′; antisense strand: 5′5′UUAUUGUCAAAUGUGAUCAGG-3′),

(#3) Rn_LOC288371_2 (sense strand: 5′-ACGAGAUCCCUGACUGUUATT-3′; antisense strand: 5′-UAACAGUCAGGGAUCUCGUTG-3′). Only siRNA #2 efficiently reduced TRPML1 expression in primary motor neurons.

In NSC-34 cells TRPML1 knocking-down was achieved by the following FlexiTube siRNAs (Qiagen, Milan, Italy) (all at 10 nM):

(#1) Mm_Mcoln1_2 (sense strand: 5′-GAUCACAUUUGACAAUAAATT-3′; antisense strand: 5′-UUUAUUGUCAAAUGUGAUCAG-3′),

(#2) Mm_Mcoln1_5 (sense strand: 5′-CAAGAACCUCACACUGAAATT-3′; antisense strand: 5′-UUUCAGUGUGAGGUUCUUGTA-3′). In NSC-34 cells, TRPML1 protein expression was successfully reduced only by using siRNA #2.

### Western blotting and immunoprecipitation

Cells were lysed by using an ice-cold buffer containing 20 mM Tris-HCl (pH 7.5), 10 mM NaF, 1 mM phenylmethylsulfonyl fluoride, 1% NONIDET P-40, 1 mM Na_3_VO_4,_ and a protease inhibitor mixture (Roche Diagnostics). After centrifugation (13,000 rpm, 4 °C, 30 min), the supernatants were collected. Protein concentration of each sample was calculated by the Bradford assay^69^. Proteins were separated on 10% sodium dodecyl sulphate-polyacrylamide gels for TRPML1, LAMP1, LAMP2, p-AMPKα, AMPKα, beclin 1, p62, GRP78, and caspase 9 expression, and on 15% sodium dodecyl sulphate-polyacrylamide gels for LC3 expression, and then electrotransferred onto 0.2 μm nitrocellulose membranes (GE Healthcare, Milan, Italy). Then, membranes were incubated with the following antibodies: anti-TRPML1 (1:1000), anti-LAMP1 (1:1000), anti-LAMP2 (1:1000), anti-p-AMPKα (1:500), anti-AMPKα (1:1000), anti-beclin 1 (1:1000), anti-p62 (1:1000), anti-LC3 (1:1000), anti-GRP78 (1:1000), anti-caspase 9 (1:1000), and anti-α-tubulin (1:2000).

For the immunoprecipitation, cells were homogenized in a lysis buffer containing: 50 mM HEPES, 100 mM NaCl, 1.5 mM MgCl_2_, 1 mM PMSF, 0.2% Nonidet P-40, 5 μg/ml aprotinin, 10 μg/ml leupeptin, and 2 μg/ml pepstatin. Lysates were then cleared by centrifugation and immunoprecipitated (500 μg) with anti-STIM1 (1:200), or anti-TRPML1 (1:200) antibodies. After separation by SDS-PAGE, immunoblot analysis was performed by using anti-TRPML1 (1:1000), or anti-STIM1 (1:1000) antibodies, respectively.

### Cell death analysis

Cell survival/death was assessed as mitochondrial activity by the 3[4,5-dimethylthiazol-2-yl]-2,5-diphenyl-tetrazolium bromide (MTT) assay. After treatments, neurons were incubated with MTT for 1 h at 37 °C. Then, samples were collected in dimethyl sulfoxide (DMSO) and measured by using a spectrophotometer at 540 nm. The data were expressed as a percentage of cell viability compared to control cultures.

### Statistical analysis

Data are expressed as mean ± S.E.M. Statistical analysis was performed with unpaired t-test or one-way analysis of variance followed by Newman-Keuls test. Statistical significance was accepted at the 95% confidence level (p < 0.05).

## Supplementary information

Supplementary Figures

## References

[1] Chou, S. M. Pathology-light microscopy of amyotrophic lateral sclerosis in *Handbook of Amyotrophic Lateral Sclerosis* (ed. Smith, R. A.) 133–181 (Marcel Deckker Inc., 1992).

[2] Rowland LP, Shneider NA (2001). Amyotrophic lateral sclerosis. N. Engl. J. Med..

[3] Rotunno MS, Bosco DA (2013). An emerging role for misfolded wild-type SOD1 in sporadic ALS pathogenesis. Front. Cell. Neurosci..

[4] Rosen DR (1993). Mutations in Cu/Zn superoxide dismutase gene are associated with familial amyotrophic lateral sclerosis. Nature..

[5] Neumann M (2006). Ubiquitinated TDP-43 in frontotemporal lobar degeneration and amyotrophic lateral sclerosis. Science..

[6] Kwiatkowski TJ (2009). Mutations in the FUS/TLS gene on chromosome 16 cause familial amyotrophic lateral sclerosis. Science..

[7] Fecto F (2011). SQSTM1 mutations in familial and sporadic amyotrophic lateral sclerosis. Arch. Neurol..

[8] Maruyama H (2010). Mutations of optineurin in amyotrophic lateral sclerosis. Nature.

[9] Matsumoto G, Shimogori T, Hattori N, Nukina N (2015). TBK1 controls autophagosomal engulfment of polyubiquitinated mitochondria through p62/SQSTM1 phosphorylation. Hum. Mol. Genet..

[10] Nishimura AL (2004). A mutation in the vesicle-trafficking protein VAPB causes late-onset spinal muscular atrophy and amyotrophic lateral sclerosis. Am. J. Hum. Genet..

[11] Johnson JO (2010). Exome sequencing reveals VCP mutations as a cause of familial ALS. Neuron..

[12] Deng HX (2011). Mutations in UBQLN2 cause dominant X-linked juvenile and adult-onset ALS and ALS/dementia. Nature..

[13] Hadano S (2001). A gene encoding a putative GTPase regulator is mutated in familial amyotrophic lateral sclerosis 2. Nat. Genet..

[14] Skibinski G (2005). Mutations in the endosomal ESCRTIII-complex subunit CHMP2B in frontotemporal dementia. Nat. Genet..

[15] Puls I (2003). Mutant dynactin in motor neuron disease. Nat. Genet..

[16] Wu CH (2012). Mutations in the profilin1 gene cause familial amyotrophic lateral sclerosis. Nature..

[17] Chow CY (2009). Deleterious variants of Fig. 4, a phosphoinositide phosphatase, in patients with ALS. Am. J. Hum. Genet..

[18] DeJesus-Hernandez M (2011). Expanded GGGGCC hexanucleotide repeat in noncoding region of C9ORF72 causes chomosome 9p-linked FTD and ALS. Neuron..

[19] McGuire, V. & Nelson, L. M. Epidemiology of ALS in: *Amyotrophic Lateral Sclerosis* (eds Mitsumoto, H., Przedborski, S. & Gordon, P. H.) 17–41 (Taylor & Francis, 2006).

[20] Murch SJ, Cox PA, Banack SA, Steele JC, Sacks OW (2004). Occurrence of beta-methylamino-l-alanine (BMAA) in ALS/PDC patients from Guam. Acta Neurol. Scand..

[21] Blokhuis AM, Groen EJN, Koppers M, van den Berg LH, Pasterkamp RJ (2013). Protein aggregation in amyotrophic lateral sclerosis. Acta Neuropathol..

[22] Edens BM, Miller N, Ma YC (2016). Impaired Autophagy and Defective Mitochondrial Function: Converging Paths on the Road to Motor Neuron Degeneration. Front. Cell Neurosci..

[23] Renton AE, Chiò A, Traynor BJ (2014). State of play in amyotrophic lateral sclerosis genetics. Nat. Neurosci..

[24] Le Ber I (2013). SQSTM1 mutations in French patients with frontotemporal dementia or frontotemporal dementia with amyotrophic lateral sclerosis. JAMA Neurol..

[25] King A (2013). Mixed tau, TDP-43 and p62 pathology in FTLD associated with a C9ORF72 repeat expansion and p.Ala239Th MAPT (tau) variant. Acta Neuropathol..

[26] Kitamura A (2014). Dysregulation of the proteasome increases the toxicity of ALS-linked mutant SOD1. Genes Cells..

[27] Vidal RL, Matus S, Bargsted L, Hetz C (2014). Targeting autophagy in neurodegenerative diseases. Trends Pharmacol. Sci..

[28] Hu Z, Yang B, Mo X, Xiao H (2015). Mechanism and Regulation of Autophagy and Its Role in Neuronal Diseases. Mol Neurobiol..

[29] Wang IF (2012). Autophagy activators rescue and alleviate pathogenesis of a mouse model with proteinopathies of the TAR DNA-binding protein 43. Proc. Natl. Acad. Sci. USA.

[30] Barmada SJ (2014). Autophagy induction enhances TDP43 turnover and survival in neuronal ALS models. Nat. Chem. Biol..

[31] Lee JK, Shin JH, Lee JE, Choi EJ (2015). Role of autophagy in the pathogenesis of amyotrophic lateral sclerosis. Biochim. Biophys. Acta..

[32] Castillo K (2013). Trehalose delays the progression of amyotrophic lateral sclerosis by enhancing autophagy in motoneurons. Autophagy..

[33] Dong XP (2008). The type IV mucolipidosis-associated protein TRPML1 is an endolysosomal iron release channel. Nature..

[34] Wong CO, Li R, Montell C, Venkatachalam K (2012). Drosophila TRPML is required for TORC1 activation. Curr. Biol..

[35] Bargal R (2000). Identification of the gene causing mucolipidosis type IV. Nat. Genet..

[36] Sun M (2000). Mucolipidosis type IV is caused by mutations in a gene encoding a novel transient receptor potential channel. Hum. Mol. Genet..

[37] Bassi MT (2000). Cloning of the gene encoding a novel integral membrane protein, mucolipidin-and identification of the two major founder mutations causing mucolipidosis type IV. Am. J. Hum. Genet..

[38] Zhang X (2016). MCOLN1 is a ROS sensor in lysosomes that regulates autophagy. Nat. Commun..

[39] Cao Q, Yang Y, Zhong XZ, Dong XP (2017). The lysosomal Ca^2+^ release channel TRPML1 regulates lysosome size by activating calmodulin. J. Biol. Chem..

[40] Medinas DB (2018). Endoplasmic reticulum stress leads to accumulation of wild-type SOD1 aggregates associated with sporadic amyotrophic lateral sclerosis. Proc. Natl. Acad. Sci. USA.

[41] Petrozziello T (2017). ApoSOD1 lacking dismutase activity neuroprotects motor neurons exposed to beta-methylamino-L-alanine though the ^2+^ /Akt/ERK1/2 prosurvival pathway. Cell Death Differ..

[42] Penny CJ, Kilpatrick BS, Eden ER, Patel S (2015). Coupling acidic organelles with the ER though Ca^2+^ microdomains at membrane contact sites. Cell Calcium..

[43] Shen D (2012). Lipid storage disorders block lysosomal trafficking by inhibiting a TRP channel and lysosomal calcium release. Nat. Commun..

[44] Garrity AG (2016). The endoplasmic reticulum, not the pH gradient, drives calcium refilling of lysosomes. Elife..

[45] Curcio-Morelli C (2010). Macroautophagy is defective in mucolipin-1-deficient mouse neurons. Neurobiol. Dis..

[46] Medina DL, Ballabio A (2015). Lysosomal calcium regulates autophagy. Autophagy..

[47] Rashid HO, Yadav RK, Kim HR, Chae HJ (2015). ER stress: Autophagy induction, inhibition and selection. Autophagy..

[48] Sirabella R (2009). Anoxia-induced NF-kappaB-dependent upregulation of NCX1 contributes to Ca^2+^ refilling into endoplasmic reticulum in cortical neurons. Stroke..

[49] Kawamata H (2014). Abnormal intracellular calcium signaling and SNARE-dependent exocytosis contributes to SOD1G93A astrocyte-mediated toxicity in amyotrophic lateral sclerosis. J. Neurosci..

[50] Nguyen Dao K.H., Thombre Ravi, Wang Jiou (2019). Autophagy as a common pathway in amyotrophic lateral sclerosis. Neuroscience Letters.

[51] Peters OM, Ghasemi M, Brown RH (2015). Emerging mechanisms of molecular pathology in ALS. J. Clin. Invest..

[52] Bosco DA (2010). Wild-type and mutant SOD1 share an aberrant conformation and a common pathogenic pathway in ALS. Nat. Neurosci..

[53] Seibenhener ML (2004). Sequestosome 1/p62 is a polyubiquitin chain binding protein involved in ubiquitin proteasome degradation. Mol. Cell. Biol..

[54] Mitsui S (2018). Systemic overexpression of SQSTM1/p62 accelerates disease onset in a SOD1H46R-expressing ALS mouse model. Mol. Brain..

[55] Hetz C (2009). XBP-1 deficiency in the nervous system protects against amyotrophic lateral sclerosis by increasing autophagy. Genes Dev..

[56] Kang R, Zeh HJ, Lotze MT, Tang D (2011). The Beclin 1 network regulates autophagy and apoptosis. Cell Death Differ..

[57] Morimoto N (2007). Increased autophagy in transgenic mice with a G93A mutant SOD1 gene. Brain Res..

[58] Fleming A, Noda T, Yoshimori T, Rubinsztein DC (2011). Chemical modulators of autophagy as biological probes and potential therapeutics. Nat. Chem. Biol..

[59] Hara T (2006). Suppression of basal autophagy in neural cells causes neurodegenerative disease in mice. Nature..

[60] Wang QJ (2006). Induction of autophagy in axonal dystrophy and degeneration. J. Neurosci..

[61] Rudnick ND (2017). Distinct roles for motor neuron autophagy early and late in the SOD1G93A mouse model of ALS. Proc. Natl. Acad. Sci. USA.

[62] Cashman N (1992). Neuroblastoma x spinal cord (NSC) hybrid cell lines resemble developing motor neurons. Dev. Dyn..

[63] Graber DJ, Harris BT (2013). Purification and culture of spinal motor neurons from rat embryos. Cold Spring Harb. Protoc..

[64] Scorziello A (2013). NCX3 regulates mitochondrial Ca(^2+^) handling though the AKAP121-anchored signaling complex and prevents hypoxia-induced neuronal death. J. Cell. Sci..

[65] Secondo A (2018). Na^+^/Ca^2+^ exchanger 1 on nuclear envelope controls PTEN/Akt pathway via nucleoplasmic Ca^2+^ regulation during neuronal differentiation. Cell Death Discov..

[66] Secondo A (2007). BHK cells transfected with NCX3 are more resistant to hypoxia followed by reoxygenation than those transfected with NCX1 and NCX2: possible relationship with mitochondrial membrane potential. Cell Calcium..

[67] Grynkiewicz G, Poenie M, Tsien RY (1985). A new generation of Ca^2+^ indicators with greatly improved fluorescence properties. J. Biol. Chem..

[68] Urbanczyk J, Chernysh O, Condrescu M, Reeves JP (2006). Sodium-calcium exchange does not require allosteric calcium activation at high cytosolic sodium concentrations. J. Physiol..

[69] Bradford MM (1976). A rapid and sensitive method for the quantification of microgram quantities of protein utilizing the principle of protein-dye binding. Ann. Biochem..

