# Supplementary material for: The activation of Mucolipin TRP channel 1 (TRPML1) protects motor neurons from L-BMAA neurotoxicity by promoting autophagic clearance
Source: Sci Rep. 2019 Jul 24;9:10743. doi: 10.1038/s41598-019-46708-5 (PMC6656764; doi:10.1038/s41598-019-46708-5)
Supplement: Supplementary file 1 — Supplementary Figures [file 41598_2019_46708_MOESM1_ESM.pdf]

The activation of Mucolipin TRP channel 1 (TRPML1) protects motor neurons from L-BMAA neurotoxicity by promoting autophagic clearance

Valentina Tedeschi[1], Tiziana Petrozziello[1], Maria José Sisalli[1], Francesca Boscia[1], Lorella Maria Teresa Canzoniero[2] and Agnese Secondo[1,]*

[1] Division of Pharmacology, Department of Neuroscience, Reproductive and Odontostomatological Sciences, School of Medicine, "Federico II" University of Naples, Via S. Pansini 5, Napoli, 80131, Italy

[2] Division of Pharmacology, Department of Science and Technology-DST, University of Sannio, via Port'Arsa 11, 82100, Benevento, Italy

* Correspondence: secondo@unina.it; Tel: +39-081-7463335

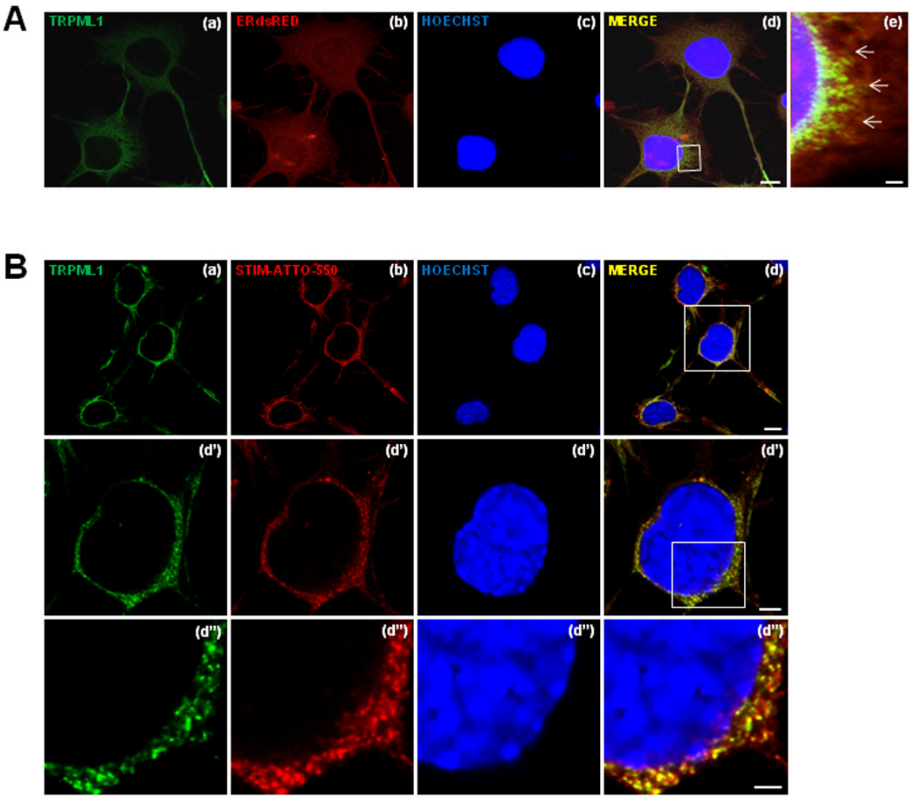

Supplementary Figure S1. (**A**) TRPML1 and ERdsRED immunosignals in differentiated NSC-34 cells (a-e). Higher magnification of the frame (e) illustrates TRPML1/ERdsRED staining in cell body. Scale bars: 10 µm (a-d), and 2 µm (e). (**B**) TRPML1 and STIM1 immunosignals in differentiated NSC-34 cells (a-d’’). Higher magnifications of the frames (d’) and (d’’) illustrate TRPML1/STIM1 staining in cell body. Scale bars: 10 µm (a-d), 5 µm (d’), and 2 µm (d’’).

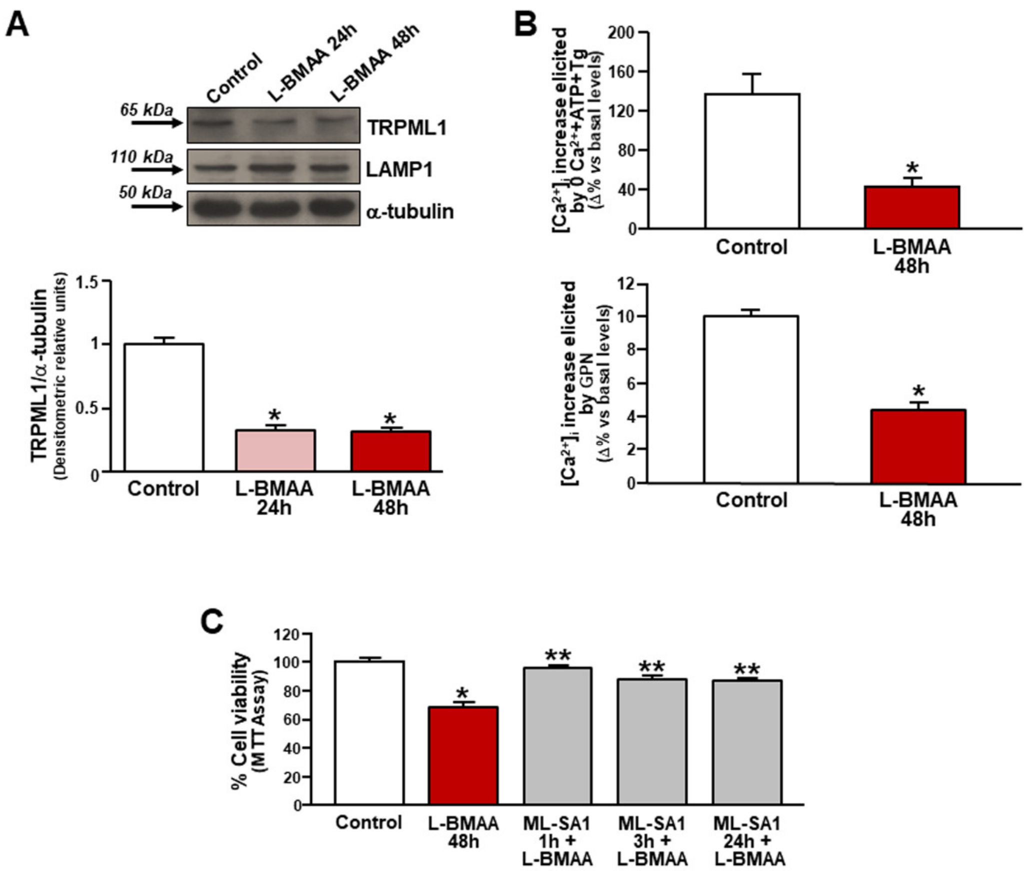

Supplementary Figure S2. (**A**) Western blotting of TRPML1 and LAMP1 expression (top) and quantification (bottom) in NSC-34 motor neurons exposed for 24 and 48 h to L-BMAA (300 μM). All experiments were repeated at least three times and expressed as mean±S.E. *p<0.05 vs control. (**B**) Bar graphs depicting the quantification of the effect of ATP (100 μM) + Tg (1 μM) in 0 Ca^{2+}(top) and of GPN (300 μM) (bottom) on $[Ca^{2+}]_i$. All experiments were repeated three times on at least 30 cells. *p<0.05 vs its respective control. (**C**) Bar graph depicting the effect of ML-SA1 (10 μM) on cell viability rate of NSC-34 cells exposed to L-BMAA (300 μM/48 h). ML-SA1 was preincubated 1 h, 3 h or 24 h before L-BMAA. Data are expressed as mean±S.E. of three different experimental sessions. *p<0.05 vs control; **p<0.05 vs L-BMAA alone.

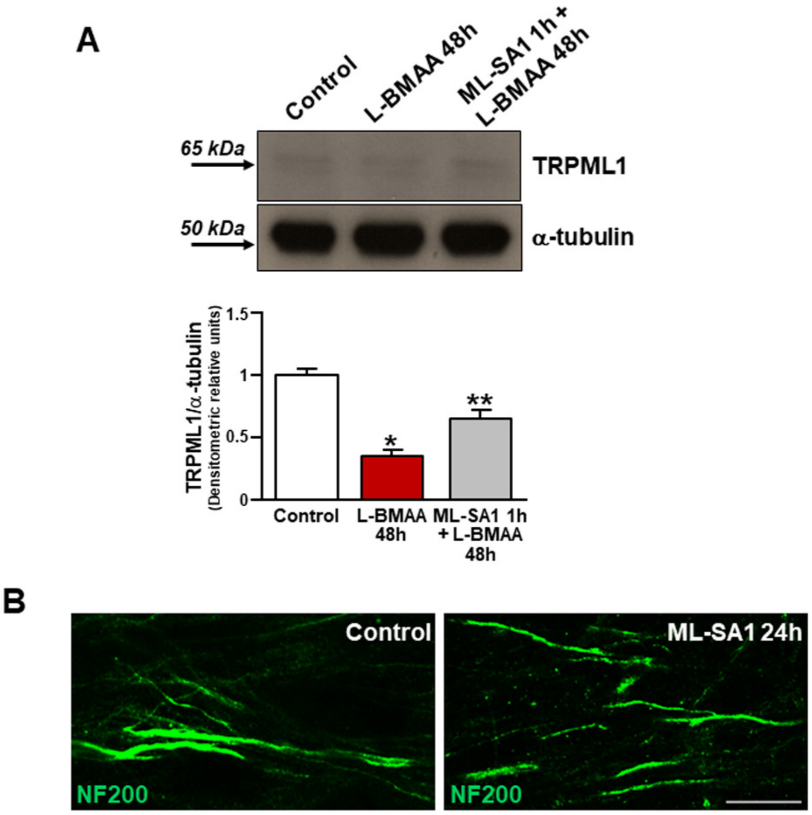

Supplementary Figure S3. (**A**) Representative Western blotting (top) and quantification (bottom) of TRPML1 expression in rat primary motor neurons exposed to L-BMAA (300 μM/48 h) in the absence or presence of ML-SA1 (10 μM). Each bar represents the mean±S.E. of data obtained from three different sessions. *$p<0.01$ vs control; **$p<0.05$ vs L-BMAA. (**B**) Representative immunocytochemical images of NF200 in rat primary motor neurons treated for 24 h with ML-SA1 alone (10 μM).
